# From Entrapment to Relief: Case Series Analysis of Advanced Ring Removal Techniques in Delayed Ring Entrapment Presentations

**DOI:** 10.7759/cureus.82896

**Published:** 2025-04-24

**Authors:** Kwabena A Danso

**Affiliations:** 1 Emergency Medicine, Komfo Anokye Teaching Hospital, Kumasi, GHA

**Keywords:** diamond cutting disc, finger ring entrapment, ring entrapment, ring removal, ring tourniquet syndrome

## Abstract

While rings serve as decorative or symbolic accessories on the hand, they can pose significant health risks when trapped on a finger. Delayed ring removal can lead to complications such as skin ulceration, neurovascular damage, and inflammation. This case series demonstrates that conventional ring removal techniques, such as compression, traction, lubrication, and rotation, are frequently ineffective when rings are deeply embedded within tissues. The GEM II ring cutter, equipped with a diamond cutting disc and a low-speed, high-torque mechanism, provides a safe and efficient solution for these challenging cases. The integrated finger guard minimizes the risk of further tissue injury, making it a valuable tool for ring removal in advanced ring entrapment or ring tourniquet syndrome.

## Introduction

While rings are mostly worn for personal adornment or as a sign of status or affiliation, others wear them for cultural or personal reasons [1]. Historically, the Egyptian pharaohs were the first to wear rings to symbolize eternity. They likened rings to the shape of a sun or moon which were their main object of worship [2]. Since then, many have adopted the wearing of rings for various reasons. Although rings are generally safe on human fingers, there seems to be more patients presenting at the Emergency Departments (EDs) with trapped rings on their fingers which cannot be ignored. A ring stuck on a finger is termed as ring entrapment. Other names include ring tourniquet syndrome [3].

Entrapment is commonly due to minor injuries and changes such as bites, hits, pregnancy and skin infections, occurring to the hand or specifically the finger that wears the ring [3,4]. This results in swelling of the finger with difficulty in moving the ring past the proximal interphalangeal (PIP) joint [3]. Persons with ring entrapment may panic and try to forcefully remove the ring, causing further swelling of the PIP joint and distal finger. The ring acts as restrictive band, reducing venous flow and eventually arterial supply to the distal structures. Without immediate presentation to an appropriate healthcare facility, this leads to soft tissue injuries with nerve and tendon damage, with the ring eventually burrowing deep to reach and expose the bone [3].

There are, however, many well-documented ways to remove entrapped rings. These include the use of lubricants such as petroleum jelly, gel, or soap solution; compression-based method where the distal finger is wrapped with an elastic band to compress swelling of the PIP joint and facilitate ring removal; and the rotation-based technique [5]. The methods mentioned are non-destructive to the rings.

However, to successfully remove an entrapped ring with significant digit swelling and/or ulceration, destructive methods must be employed. Successful and safe destructive cutting of an entrapped ring depends on knowing the width, thickness or height of the ring, as well as the metal the ring was forged from, to aid with the exact tool or cutting disc to use [6].

We present a case series of delayed finger ring entrapment, which only could be removed efficiently and effectively with the GEM II ringer cutter and its diamond cutting disc.

## Case presentation

Case 1

A male patient, late 20s, presented to the ED accompanied by his sister, with complaint of a swollen left ring finger. He was not forthcoming with information and uncertain about the exact occurrence of events that led to he having a swollen left ring finger. His sister, not living with him, recounted being called by some people about her brother’s state. Witnessing the state of his finger and with unsuccessful home attempts at ring removal, they presented to the ED.

**Figure 1:**
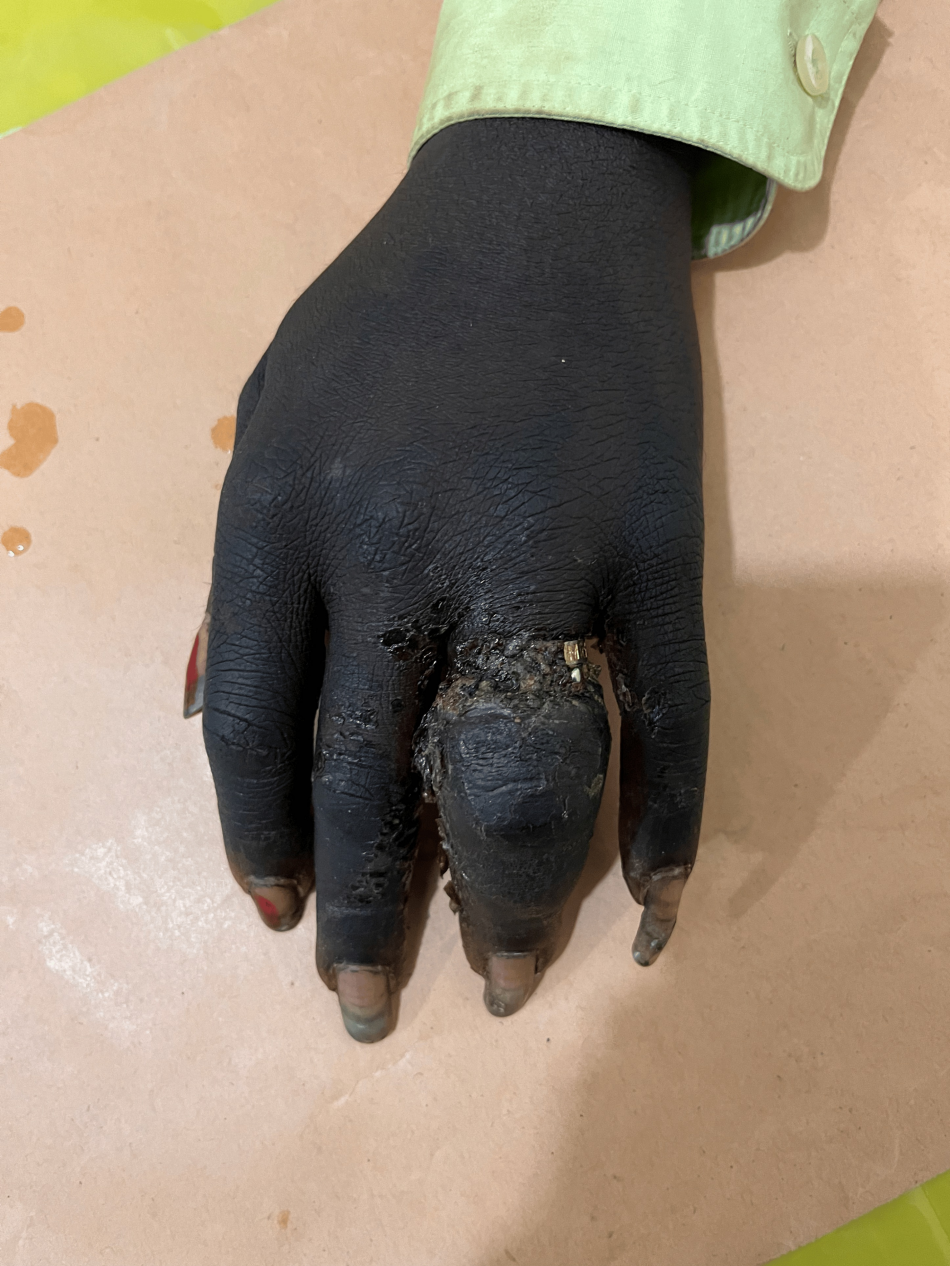
Dorsal aspect of left hand at presentation

**Figure 2:**
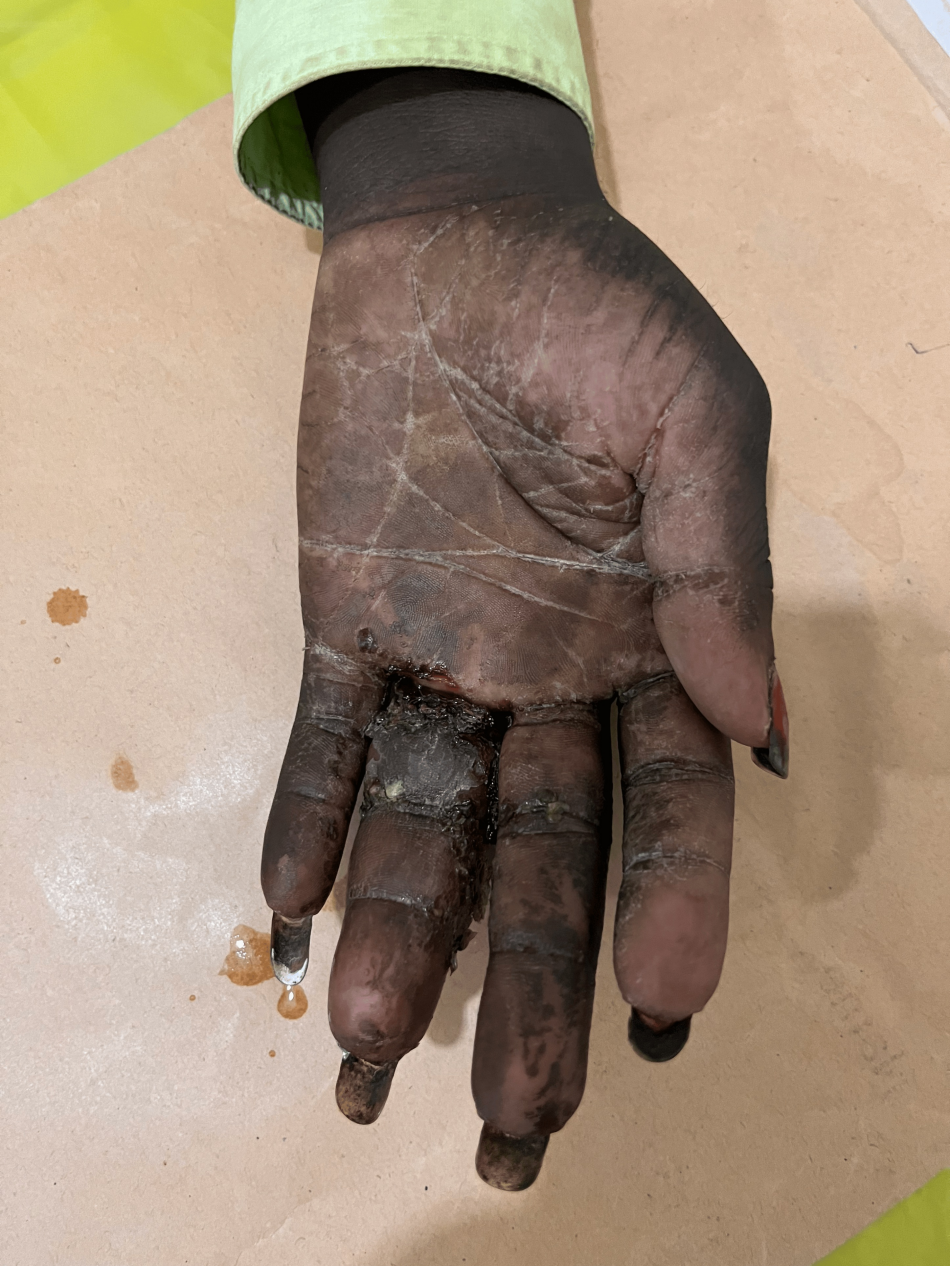
Palmar surface of left hand at presentation

**Figure 3:**
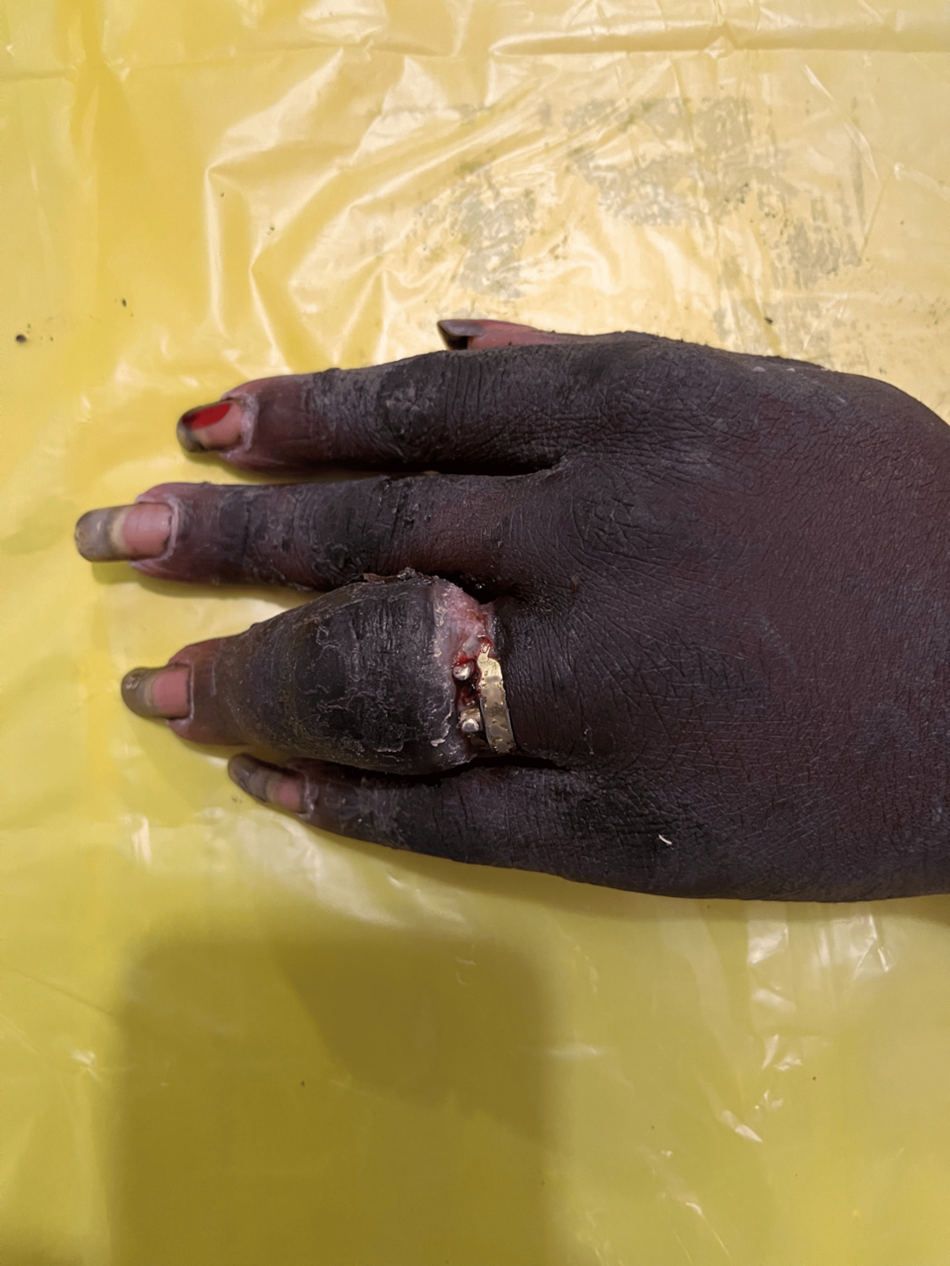
Hand after cleaning with lukewarm antiseptic solution. Two rings exposed after washing.

**Figure 4:**
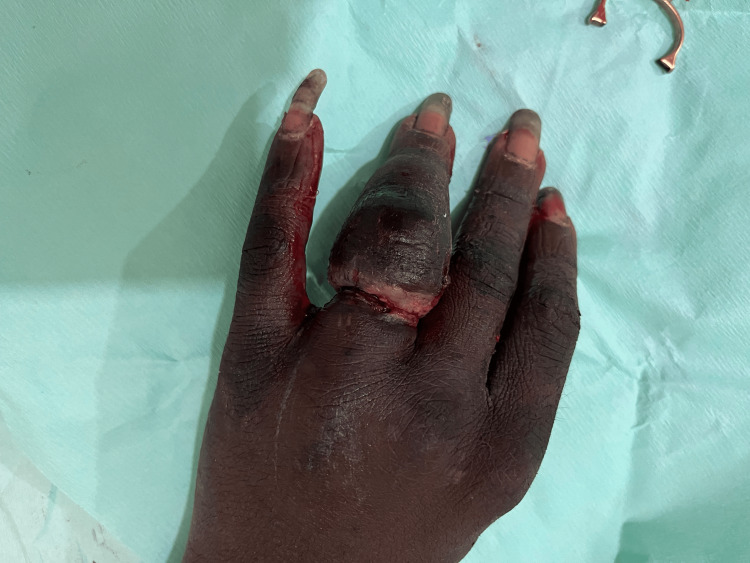
Dorsal aspect of the left hand following removal of two rings from the ring finger

On examination, the patient looked unkempt, and his left hand was visibly soiled and grossly contaminated with dirt and other organic material dorsally and ventrally, extending to the wrist (Figures 1, 2). The left ring finger was markedly swollen from the PIP joint to the distal phalanx with marked granulation tissue over the dorsal proximal phalanx, affecting visualization of the ring. There was normal sensation and capillary refill time of the involved finger, and no signs of infection. All vitals were within normal ranges. Patient was counseled in the presence of his sister for ring removal and the procedure was explained. Digital nerve block of the left ring finger was applied using 8 ml of 1% plain lidocaine, more proximal to the wound site and sensation tested again for effectiveness. The hand was soaked in lukewarm saline and antiseptic solution to thoroughly wash of all dirt and loose tissues (Figure 3). Washing exposed two separate rings, and the distal ring was easily removed using a dedicated ring spreader. The remaining ring was lubricated with water-based gel and a finger guard was gently applied underneath to prevent damage to skin and other tissues during cutting. The GEM II, four-AA-battery-powered ring cutting device with a diamond cutting disc was used to cut the thick ring at two points and open for safe removal with the ring spreader (Figure 4). The wound was cleaned and dressing applied, and the patient was asked to elevate the hand. Oral antibiotics were prescribed and the patient was scheduled for review on out-patient basis but was lost to follow-up.

Case 2

**Figure 5:**
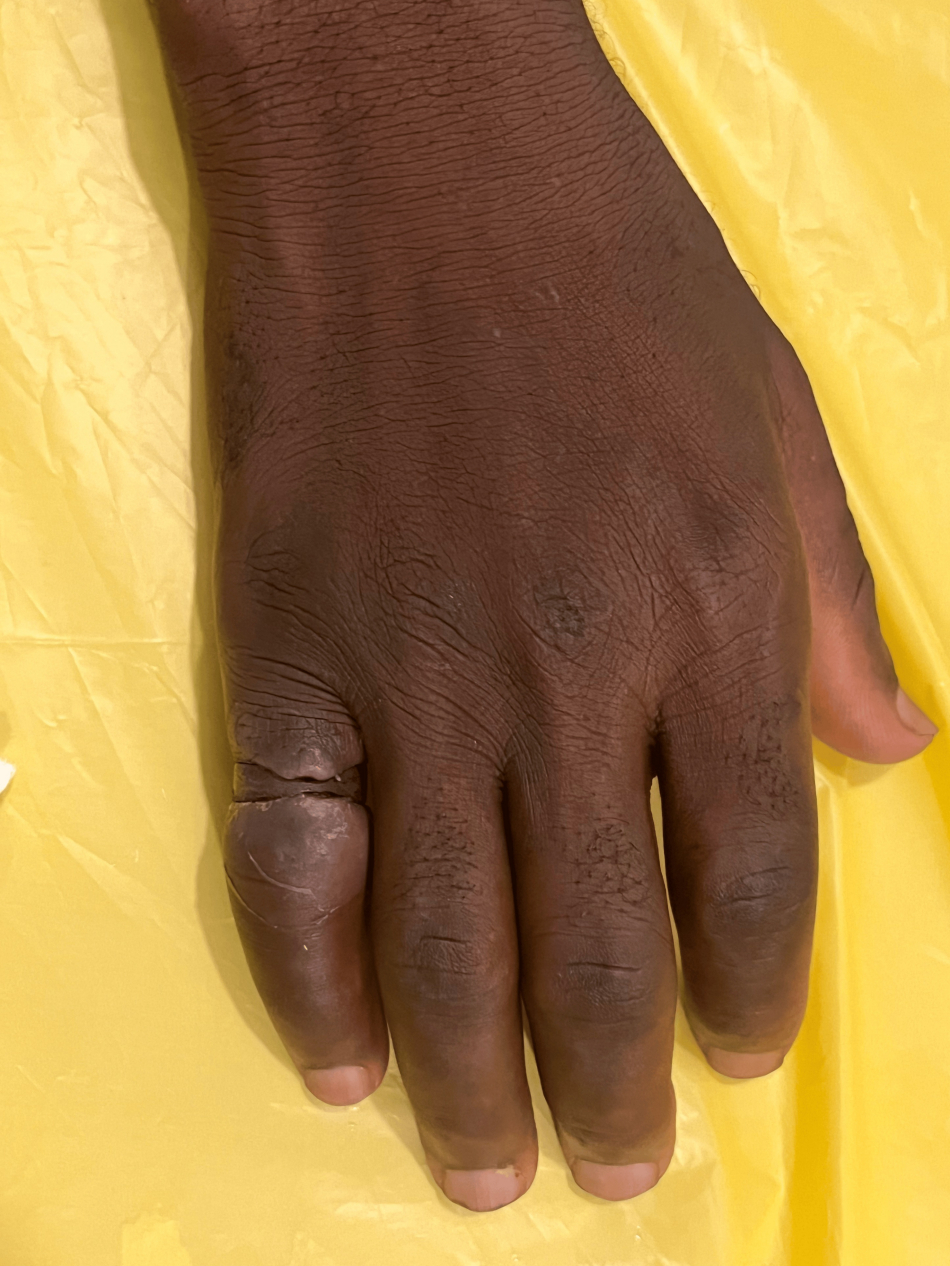
Dorsum of right hand with ring buried within skin of the little finger

A 38-year-old male patient, a mental health service user, presented with a 14-day history of pain and swelling of his right little finger. He recollected some pain but no obvious trauma, with associated difficulty in removing the ring from his little finger. He tried removing it several times with soap solution and oils but was unsuccessful. Relatives noticing the changes over the dorsum of his right fifth digit, and he decided to report to the health facility (Figure 5).

**Figure 6:**
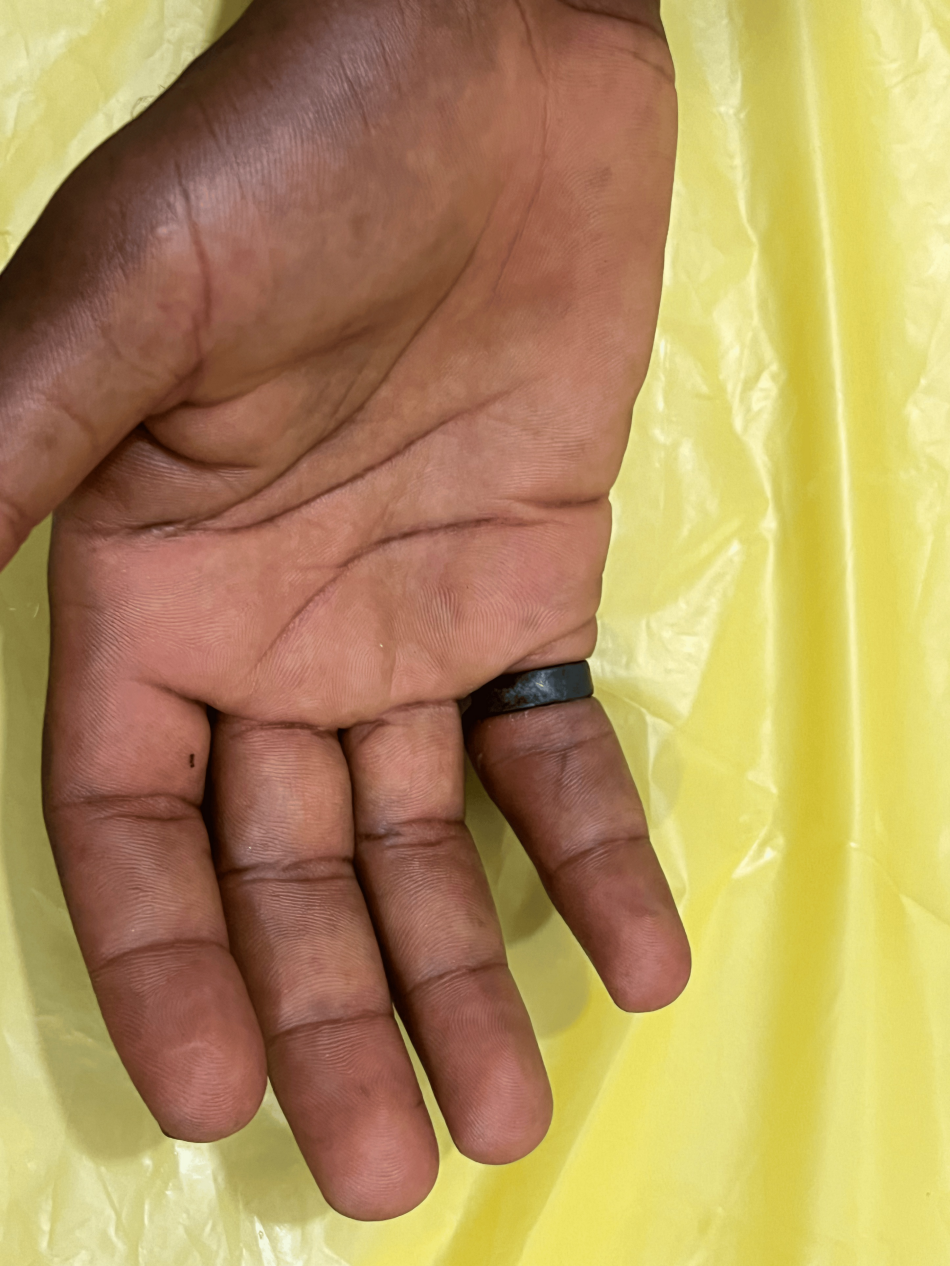
Palmar aspect of right hand showing the free ring surface without skin edema or ulceration

**Figure 7:**
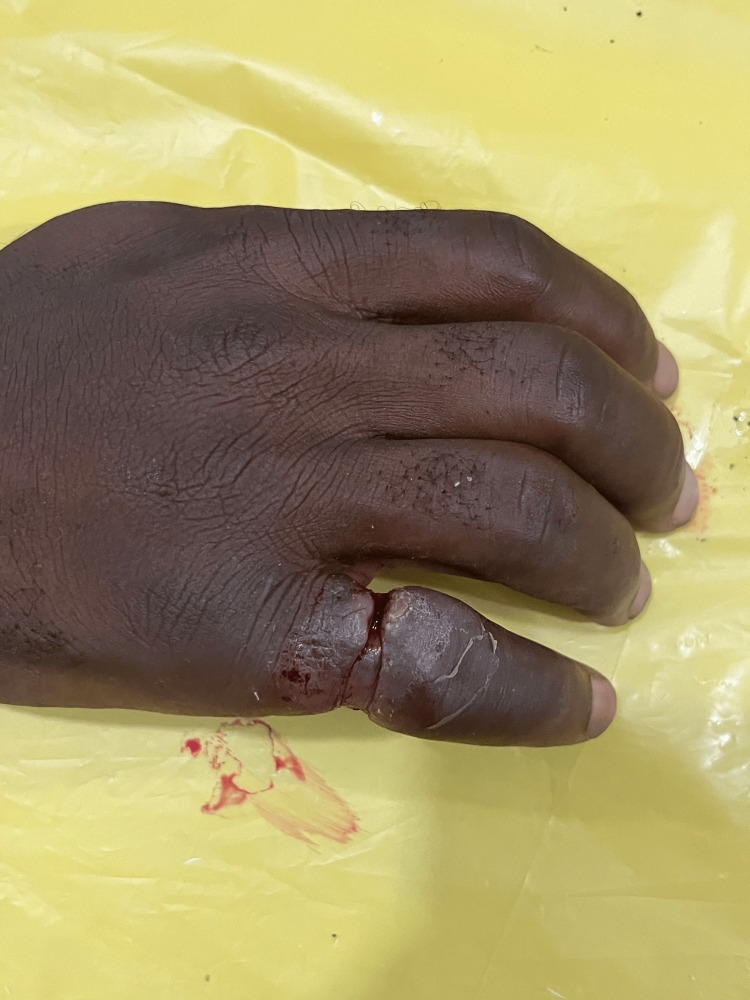
Dorsum of the right hand following ring removal from the little finger

**Figure 8:**
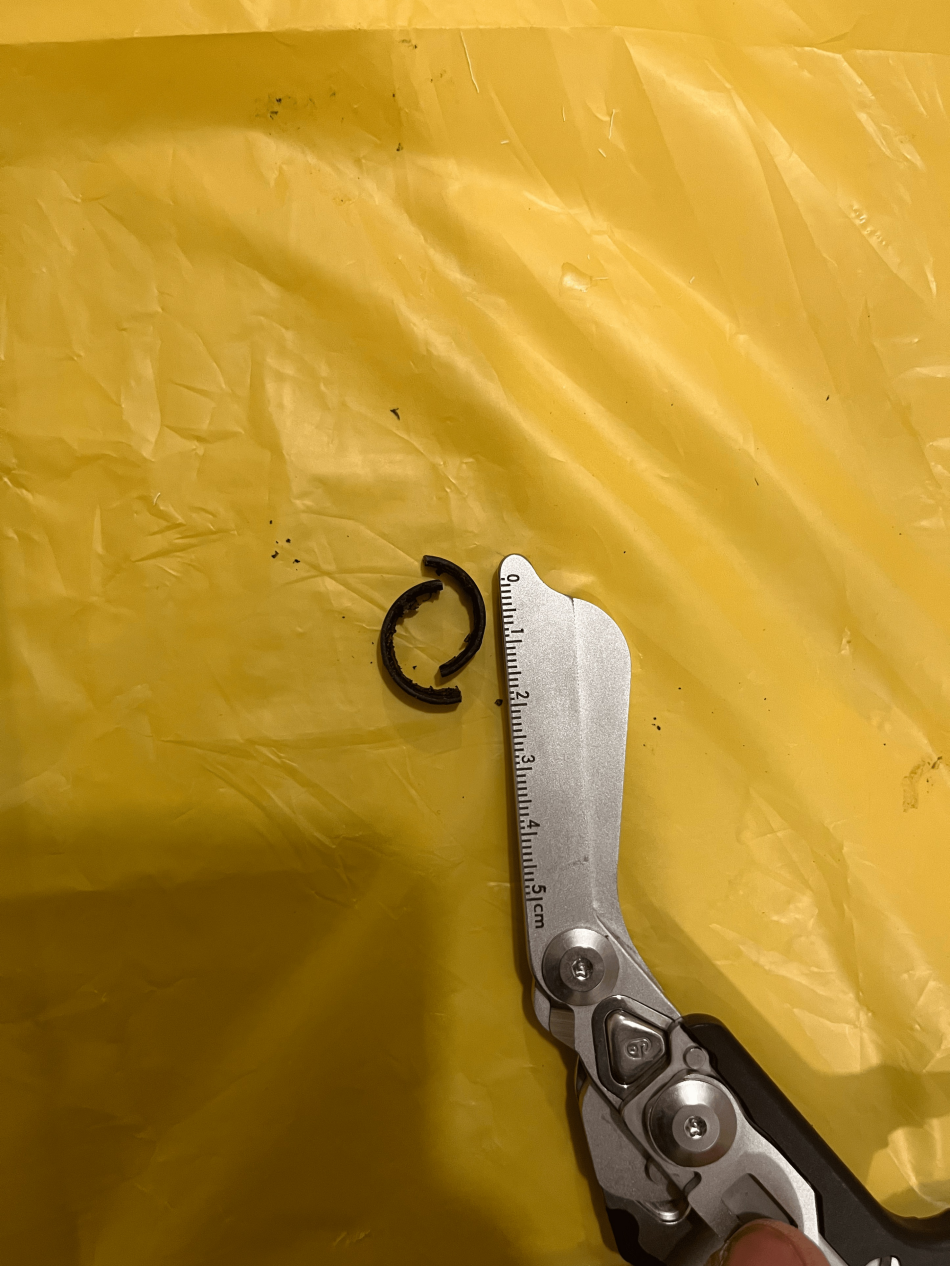
Showing ring cut in two places for easy removal

During examination, the patient, an adult male, was accompanied by relative and looked nervous and unkempt. All vital signs were within range. The condition and procedure of ring removal were explained to both the relative and the patient, and verbal consent was sought. The palmar surface of his little finger looked normal and intact with a black outside of a ring (Figure 6), whereas dorsum showed areas of peeled skin over the swollen middle phalanx and ring burrowed into the skin of the proximal phalanx. Distal sensation and flexion of the distal interphalangeal (DIP) joint were intact and capillary refill time normal. Digital nerve block with 8 ml of 1% lidocaine was given and a finger guard was placed between the ring and skin surface on the palmar aspect with aid of a water-based gel. The gel was again applied over the site of the ring to be cut, as a means to reduce frictional burns during cutting. The wide tungsten ring was cut in two places with the diamond disc of the four-AA-battery-powered GEM II ring cutter (Figures 7, 8). The site was cleaned, sterile dressing was applied, oral antibiotic were prescribed, and the patient was scheduled for review on out-patient basis.

Case 3

**Figure 9:**
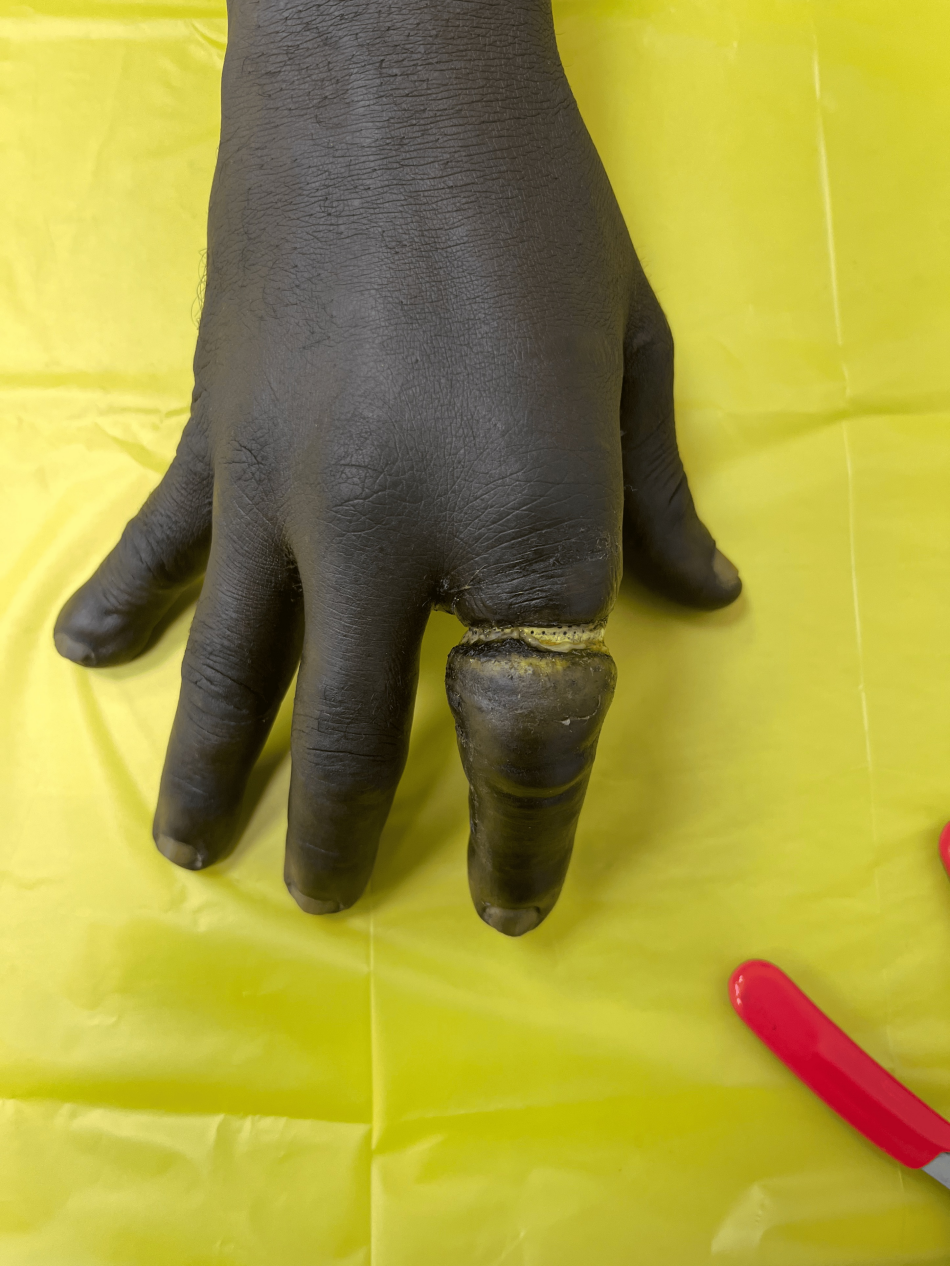
Right hand showing swelling of the proximal phalanx and interphalangeal joint of the index finger

**Figure 10:**
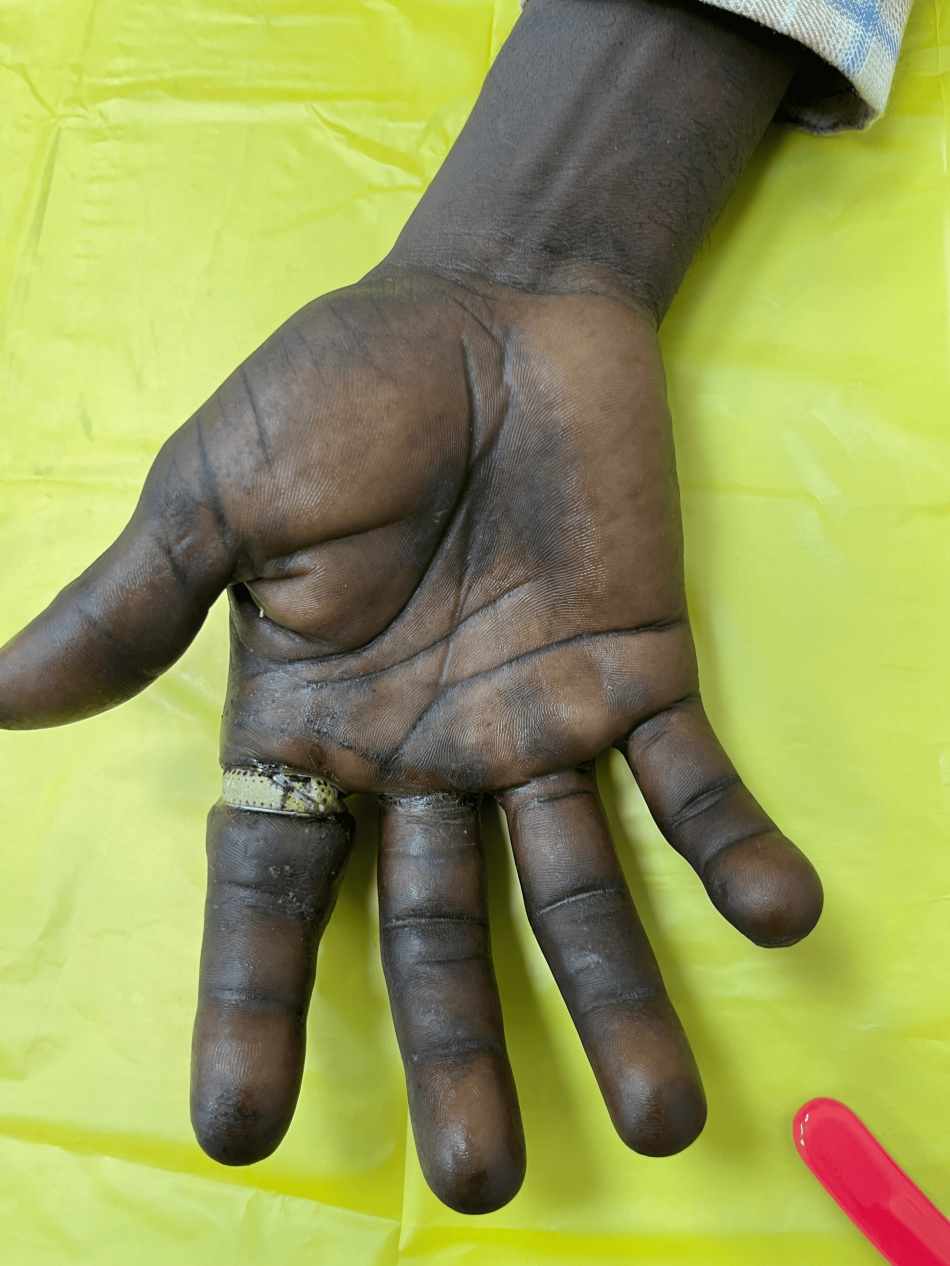
Palmar surface of the right hand with a wide width ring at the base of the index finger

A middle-aged male patient self-reported to the ED with a 10-day history of pain and swelling of the right index finger after he sustained an injury while working on his vehicle. He had multiple failed attempts at ring removal. His vital signs were within normal range. Examination of his right hand showed an edematous proximal phalanx of the index finger, with granulation tissue over the dorsal aspect along the margin where the ring meets the skin (Figures 9, 10). There was some pus-like discharge over the dorsal skin. However, capillary refill time was two seconds, distal sensation was intact, and there was no restriction to joint movement.

**Figure 11:**
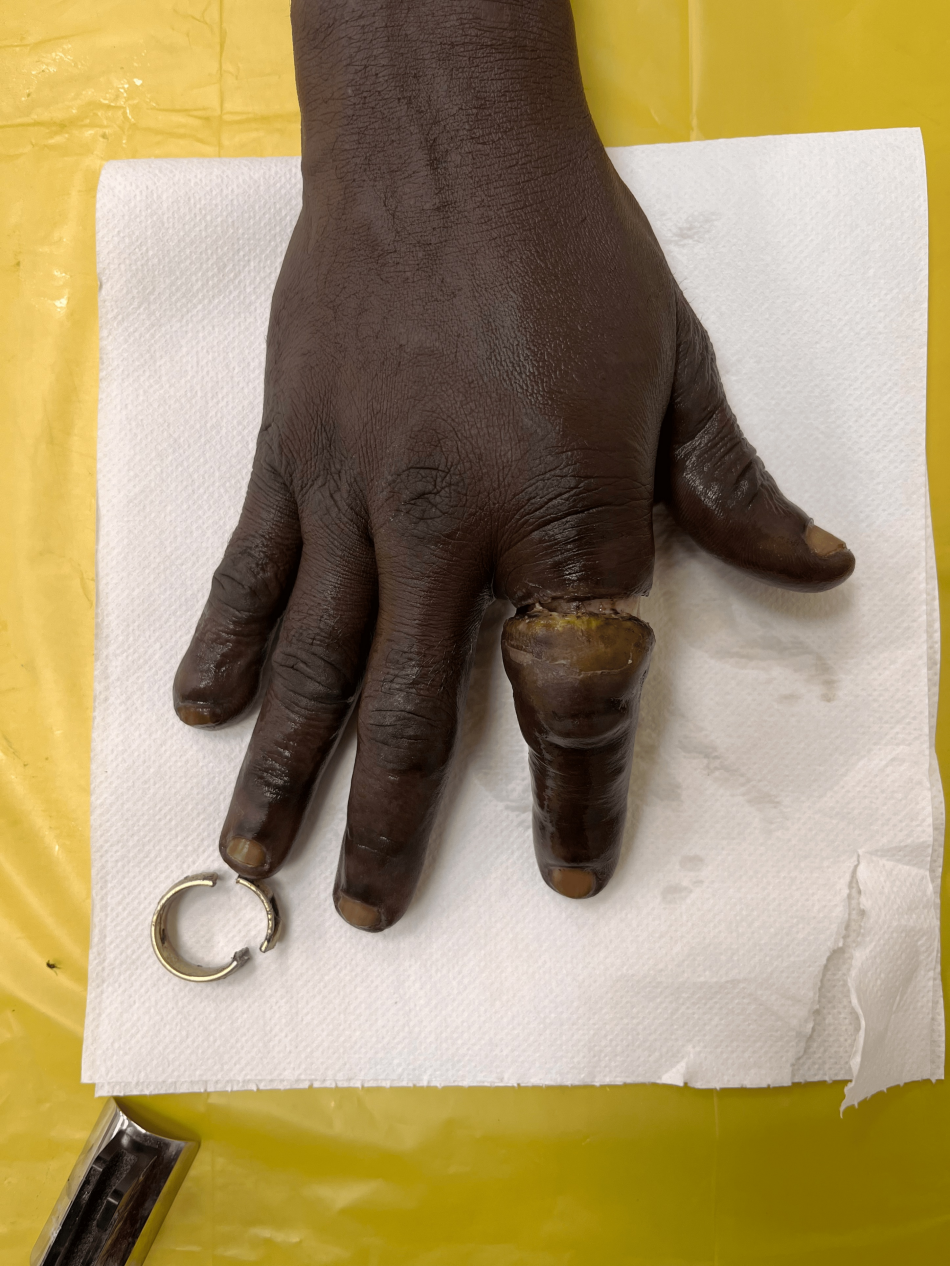
Dorsum of the right hand showing the floor of the wound after ring removal and ring cut in two places for easy removal

**Figure 12:**
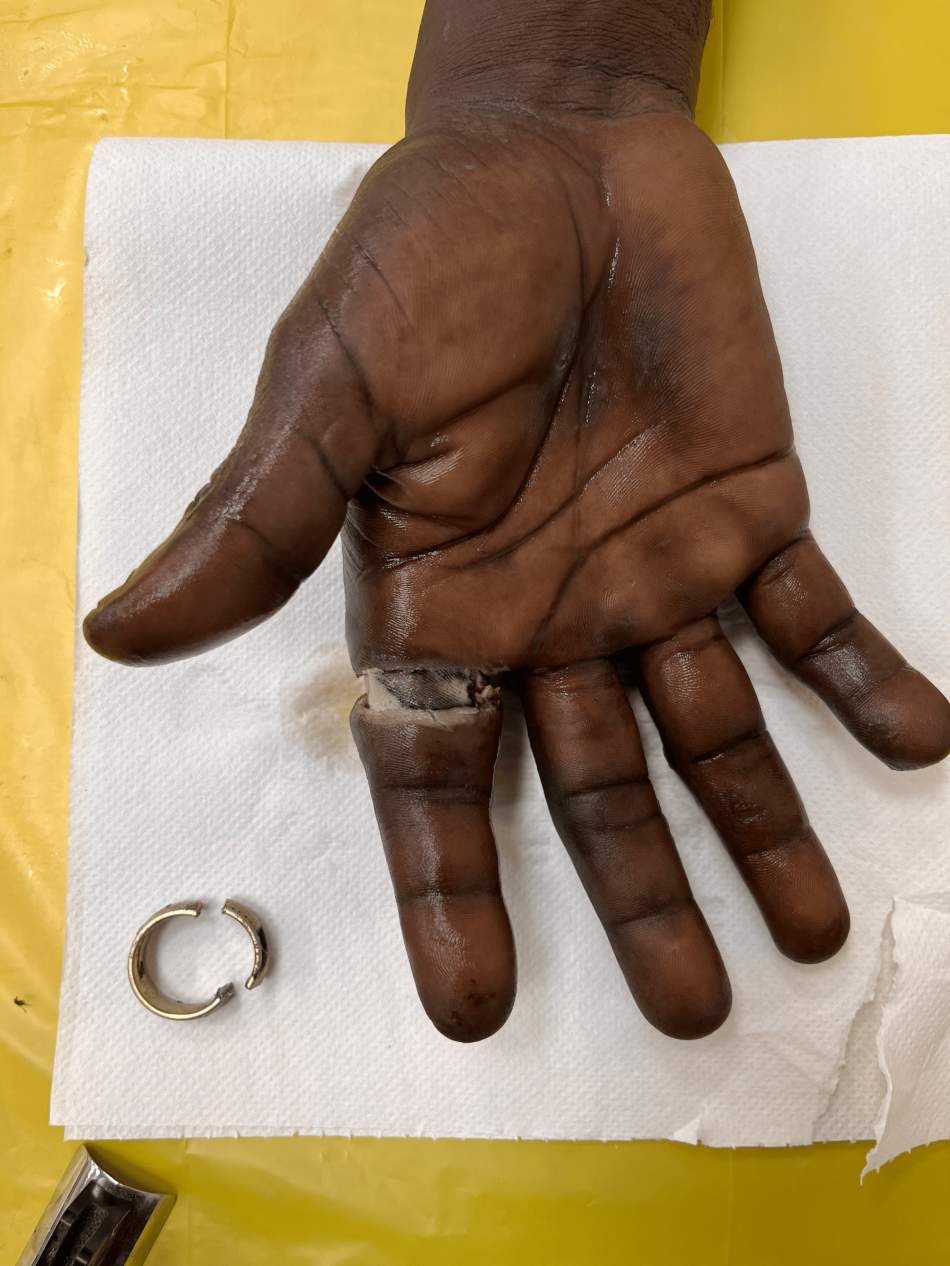
Palmar surface of the right index finger after ring removal

The procedure and removal of the ring by the cutting method was explained and consent was sought. Digital nerve block was applied with 8 ml of 1% plain lidocaine, the right hand was cleaned with soapy water, dried, and a finger guard was placed under the ring on the palmar surface. The ring was successfully cut with the diamond cutting disc of the GEM II ring cutting device (Figures 11, 12). The wound was irrigated and cleaned, and sterile dressing was applied. Oral antibiotics were prescribed. The patient was scheduled for review on out-patient basis but was lost to follow up.

Case 4

An adult male patient was brought to the ED by community folks, with swelling of his right hand and deformity of the right middle finger. Community folks mentioned the patient being homeless and likely to have an intellectual disability, as this could explain his above stated observation for over seven weeks. They had tried many ways to relieve him of the problem but were unsuccessful, and hence, he was presented to the ED for assistance. He had presented a week prior but was aggressive and refused the removal with physical threats to staff. The folks were concerned, and came back a second time.

**Figure 13:**
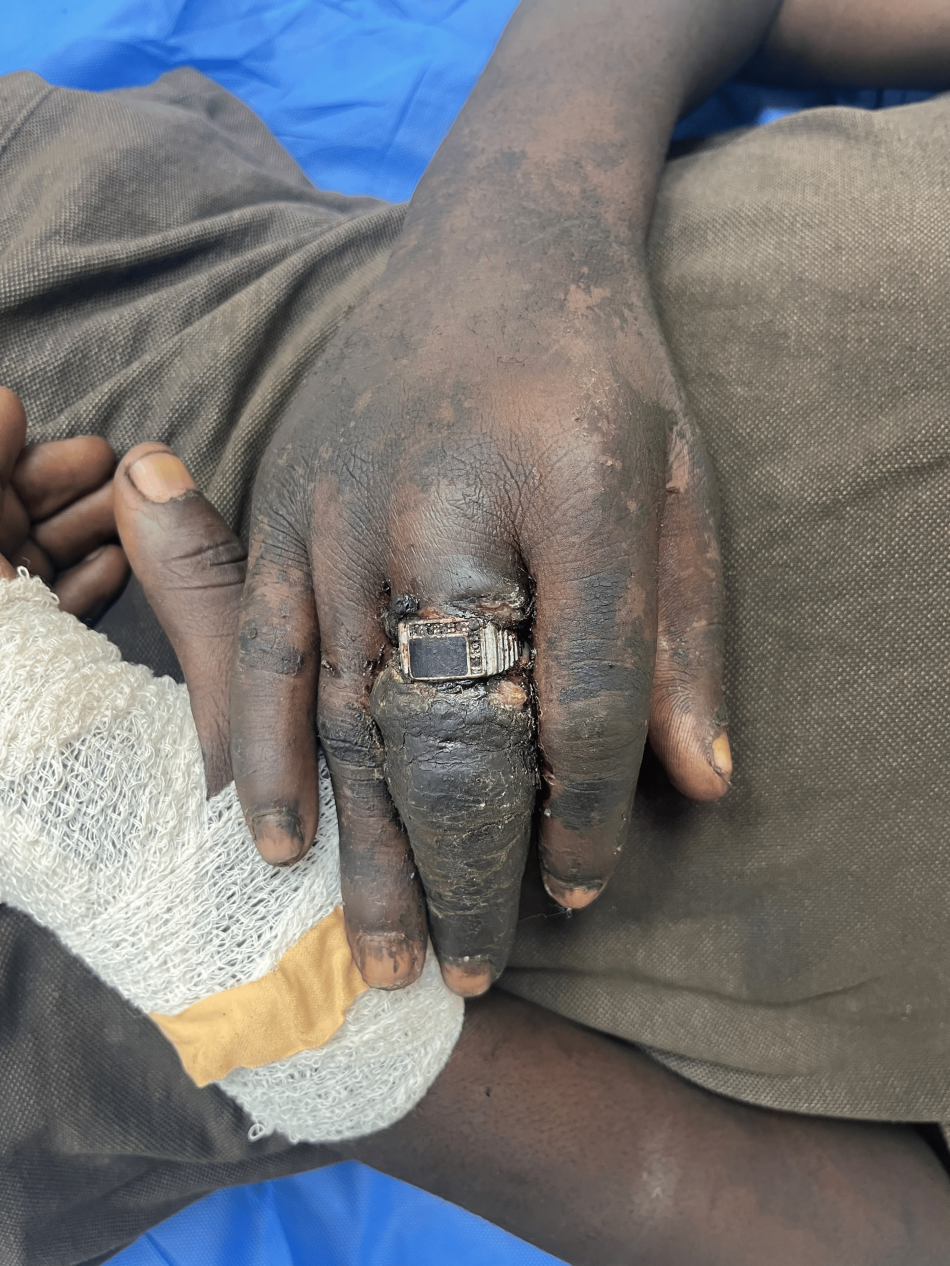
Dorsal aspect of right hand at presentation. Marked swelling of the mid and distal aspects of middle finger with visible ring

**Figure 14:**
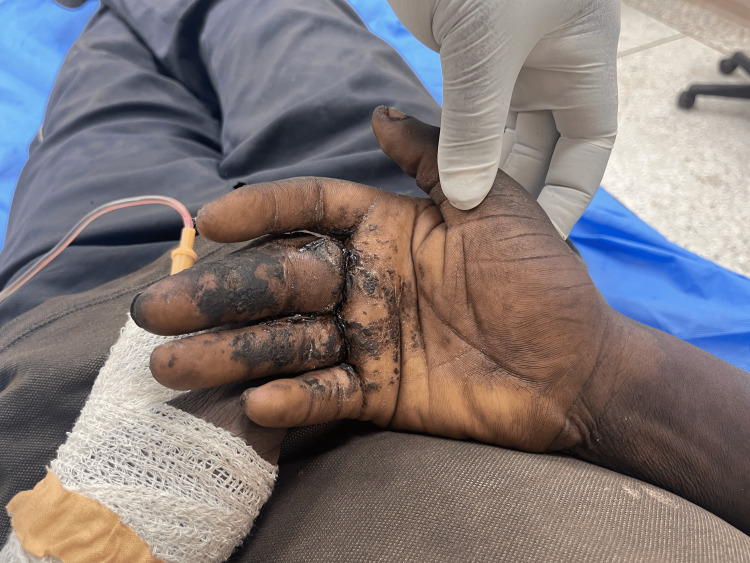
Palmar surface of the right hand with patches of dirt

**Figure 15:**
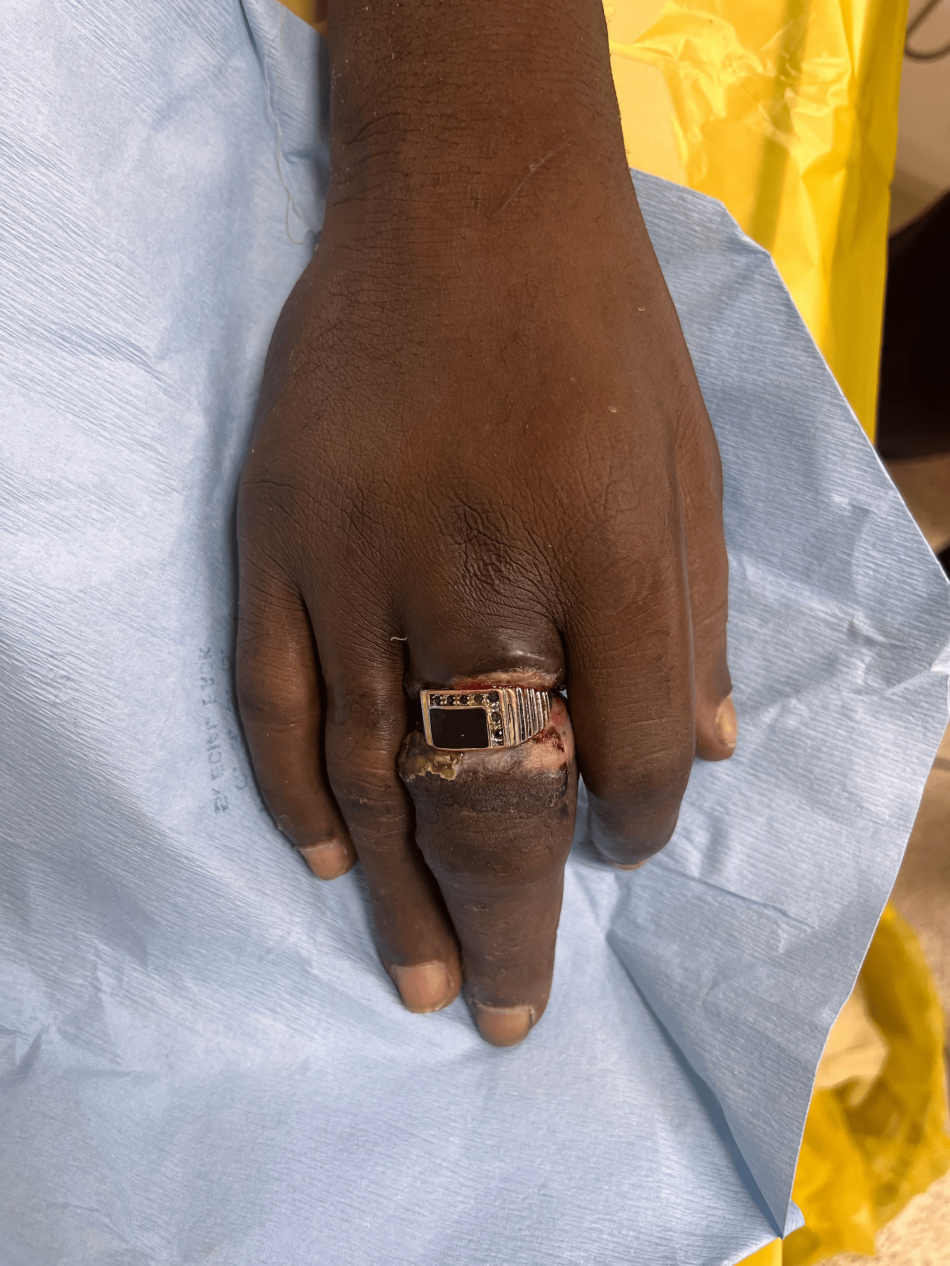
Dorsal surface of the right hand after cleaning

**Figure 16:**
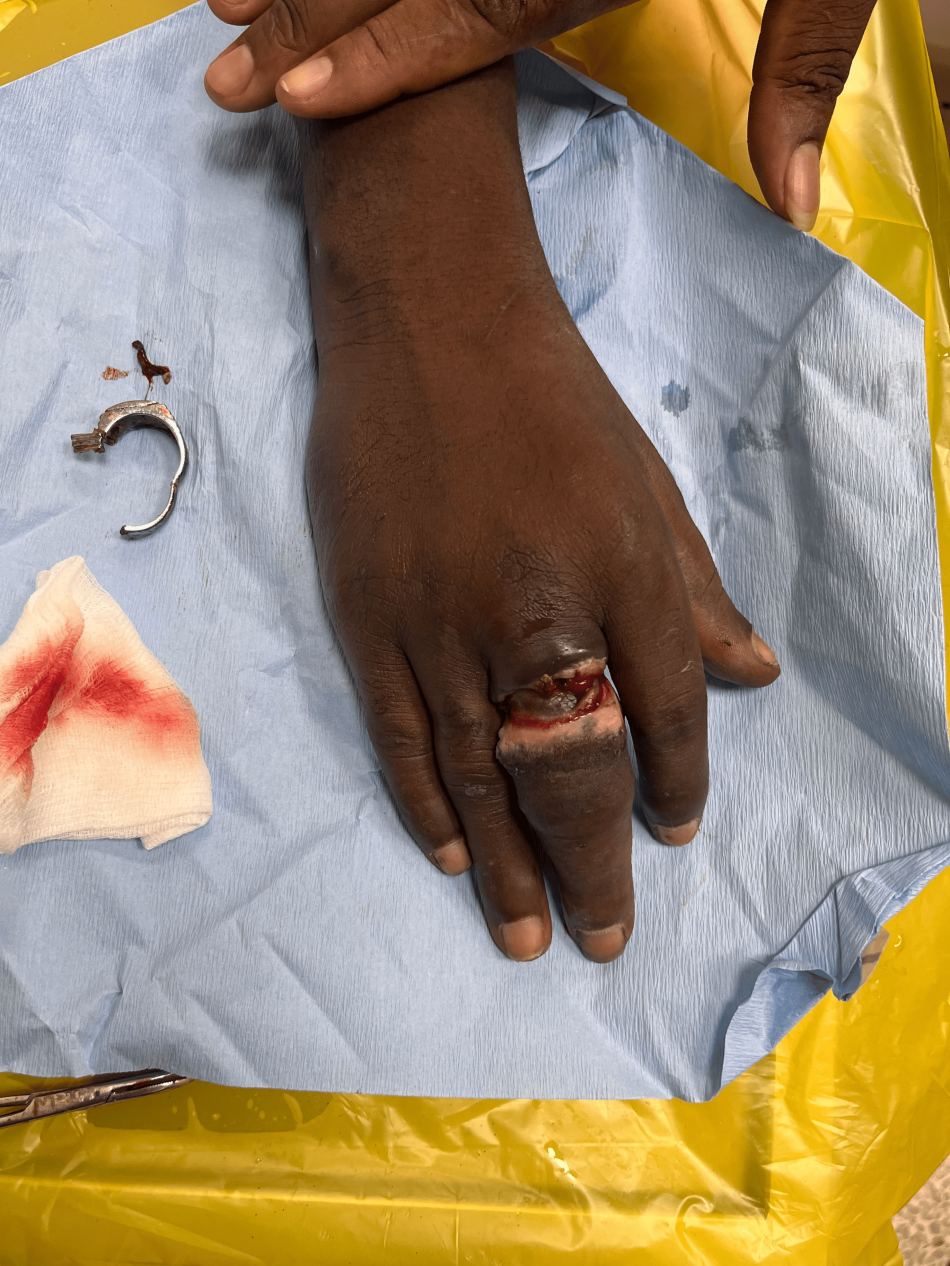
Dorsal surface of right hand after ring removal; cut ring seen on the left side of hand

The patient seen to be unkempt with dirty clothing and hands, agitated and aggressive even to his folks. Vital signs were however unremarkable. He had to be sedated for the duration of the procedure. His right hand was thoroughly washed with lukewarm tap water and antiseptic solution, and digital anesthesia was provided with 8 ml 1% plain lidocaine at the level of the distal palmar crease under ultrasound guidance (using high frequency probe). The ring was examined to aid with the best and easy approach for cutting and removal (Figures 13, 14, 15). A finger guard from the GEM II ring cutter kit was gently placed under the ring and two separate cuts were made with the diamond cutting disc. The ring was spread open to ease removal using the dedicated ring spreader. Bleeding was controlled by direct pressure and further cleaning and debridement was done (Figure 16). Sterile dressing was applied, oral antibiotics were prescribed, and the patient was scheduled for review on out- patient basis and consult for psychiatric evaluation. The patient was monitored until full recovery from sedation before discharge.

## Discussion

Rings are worn for several reasons on any of the fingers. These reasons include fashion, culture, or as a symbol of power. Ring entrapment can occur after minor injuries like hitting a hand or the finger with the ring against objects, or sustaining cuts or tears that lead to swelling. In some cases, however, the exact cause remains unknown. These events lead to swelling of the finger, especially at the PIP joint. The ring is unable to pass over the swollen joint and becomes entrapped.

The cases presented were patients reporting late to the ED with entrapped rings that threatened the integrity of their fingers, leading to swelling and reduction of venous and ultimately arterial blood supply [3]. Without immediate interventions or removal, the ring erodes the skin tissue and may lead to tendon and nerve damage, necrosis, or even amputation [7].

This paper presents four patients reporting late to the ED with entrapped rings, forged from heavy metals such as gold, titanium, and tungsten. The rings seen had been thick and wide, which made cutting with manual ring cutting devices tedious and time consuming, and could easily blunt the cutting blade without any success. Removal by methods like lubricants, traction-based techniques, and compression methods in the above cases was not recommended [4,5,7].

For the cases described above, the digits wearing the rings had significant PIP-joint edema with associated ulcerations and burrowing of the skin. Attempting ring removal with non-destructive methods, such as lubricating gel, was not feasible due to the distorted anatomy of the digit. The caterpillar technique, first used by St. Laurent in 2006, and the string pull method, first used in the 1986, both employing traction-based maneuver, would not have been appropriate for persons presenting late with ring tourniquet syndrome [5]. In these cases, degree of skin and other tissue changes of the involved fingers would not have permitted passing strings or allow the use of rocking movement to aid the safe removal of the ring. Also, attempting to compress the distal swelling with an elastic band, as described by the compression-based method, would have been unsuccessful as the rings were seen embedded deep into the skin surface [5]. It is highly recommended that a destructive approach be used in cases of late presentation with significant surrounding tissue changes as described. Another important intervention is the use of local anesthesia during the use of destructive method, preferably with plain lidocaine, as the exact vascular integrity is assessed after the procedure.

In the cases presented, the rings can be profiled as having great widths and heights and being forged from heavy metals (gold, titanium, and tungsten). The above, coupled with significant skin and tissue injury, made ring removal by the simple non-destructive ring methods ineffective. Hence, the battery-powered GEM II ring cutter, with its diamond cutting disc and protective finger guard, was used for effective yet safe ring removal. The finger guard placed between the ring and skin surface served as a protective barrier to prevent cuts and injuries to the patient while effectively sawing through the rings even when there was marked tissue damage prior to the removal process [3]. The risk of thermal injury during cutting is minimized by applying a water-soluble gel to the part of the ring being cut under the rotating diamond cutting disc.

## Conclusions

Early removal of constricting objects like rings from fingers following even minor hand or finger trauma is necessary to prevent ring entrapment or ring tourniquet syndrome. It is paramount for physicians to know the metal from which rings are forged and the profile of the rings as it is helpful to hasten removal. Late presentation of finger ring entrapment or cases with extensive damage to skin and other tissues can best be resolved by destructive measures like cutting the ring. The use of a motorized device with dedicated cutting discs for heavy and thick rings aids in immediate resolution of ring tourniquet syndrome and prevents further complications. 
